# Effects of Glycerol and Creatine Hyperhydration on Doping-Relevant Blood Parameters

**DOI:** 10.3390/nu4091171

**Published:** 2012-08-31

**Authors:** Thelma P. Polyviou, Chris Easton, Lukas Beis, Dalia Malkova, Pantazis Takas, Catherine Hambly, John R. Speakman, Karsten Koehler, Yannis P. Pitsiladis

**Affiliations:** 1 Institute of Cardiovascular & Medical Sciences, College of Medicine, Veterinary and Life Sciences, University of Glasgow, Glasgow, G12 8QQ, UK; Email: t.polyviou.1@research.gla.ac.uk (T.P.P.); l.beis.1@research.gla.ac.uk (L.B.); p.takas.1@research.gla.ac.uk (P.T.); 2 Faculty of Science, Engineering and Computing, Kingston University, Kingston upon Thames, Surrey KT1 2EE, UK; Email: C.Easton@kingston.ac.uk; 3 Medical School, College of Medicine, Veterinary and Life Sciences, University of Glasgow, Glasgow G12 8QQ, UK; Email: Dalia.Malkova@glasgow.ac.uk; 4 Institute of Biological and Environmental Sciences, University of Aberdeen, Tillydrone Ave, Aberdeen AB24 2TZ, UK; Email: c.hambly@abdn.ac.uk (C.H.); j.speakman@abdn.ac.uk (J.R.S.); 5 Institute of Biochemistry, German Research Center of Elite Sport, German Sport University Cologne, Cologne 50933, Germany; Email: koehler@dshs-koeln.de

**Keywords:** masking agent, glycerol, creatine, hyperhydration, doping markers

## Abstract

Glycerol is prohibited as an ergogenic aid by the World Anti-Doping Agency (WADA) due to the potential for its plasma expansion properties to have masking effects. However, the scientific basis of the inclusion of Gly as a “masking agent” remains inconclusive. The purpose of this study was to determine the effects of a hyperhydrating supplement containing Gly on doping-relevant blood parameters. Nine trained males ingested a hyperhydrating mixture twice per day for 7 days containing 1.0 g·kg^−1^ body mass (BM) of Gly, 10.0 g of creatine and 75.0 g of glucose. Blood samples were collected and total hemoglobin (Hb) mass determined using the optimized carbon monoxide (CO) rebreathing method pre- and post-supplementation. BM and total body water (TBW) increased significantly following supplementation by 1.1 ± 1.2 and 1.0 ± 1.2 L (BM, *P *< 0.01; TBW, *P *<0.01), respectively. This hyperhydration did not significantly alter plasma volume or any of the doping-relevant blood parameters (e.g., hematocrit, Hb, reticulocytes and total Hb-mass) even when Gly was clearly detectable in urine samples. In conclusion, this study shows that supplementation with hyperhydrating solution containing Gly for 7 days does not significantly alter doping-relevant blood parameters.

## 1. Introduction

Hyperhydration or increasing total body water (TBW) above normal is considered a prudent performance-enhancing strategy prior to strenuous exercise in the heat [1,2,3] as it leads to reduced measures of thermal strain such as attenuated core temperature (Tcore), heart rate (HR) and perception of effort during exercise in the heat [4,5]. Glycerol (Gly) combined with creatine (Cr) is an effective hyperhydration strategy resulting in significantly greater increases in TBW than when Gly is consumed alone [4]. The inclusion of Cr in the hyperhydrating supplement is crucial since Cr is known to retain fluid predominantly in the intracellular fluid compartments [6], while the addition of glucose (Glu) serves to enhance insulin release in response to a rise in blood Glu and in doing so, stimulates Cr uptake by skeletal muscle [7]. In 2010, the World Anti-Doping Agency (WADA) added Gly to the prohibited list as a potential masking agent [8] on the basis that the plasma volume (PV) expansion properties of Gly could potentially influence doping-relevant blood parameters and in doing so mask the effects of any banned substance(s).

Direct detection of blood manipulations such as use of recombinant human erythropoietin (r-HuEpo) is difficult. Therefore, recent advances in detection of doping include the development of the Athlete Biological Passport (ABP) of doping which involves monitoring of biomarkers such as hemoglobin concentration (Hb), reticulocytes (Ret%), and analysis of these markers with the use of statistical models [9]. With this information, athletes can either be sanctioned directly based on their profile or targeted with conventional doping tests [10]. Although the blood parameters measured by the anti-doping authorities to assess doping are not uniform, the unique combination of high hematocrit (Hct) and/or Hb, suppressed erythropoiesis (low Ret% count) and reduced “stimulation index” (OFF-hr model) appears better at detecting the use of erythropoietic stimulators than the use of any of these markers alone [11,12]. To date, no study has evaluated the effects of the improved hyperhydrating solution containing Gly, Cr and Glu on the indirect markers of doping. It is unlikely that the addition of Cr will diminish the potential masking abilities of Gly, as combined Cr and Gly supplementation has been found to have the same effect on PV, Hb and Hct, compared to when Gly was consumed alone [4]. Therefore, the aim of the present study was to determine the effects of an effective Gly hyperhydrating supplement consumed for 7 days on blood parameters such Ret%, OFF-hr score and hybrid algorithm (Hbmr), which are routinely used by anti-doping authorities to assess doping. As most athletes, in situation were they may be subject to testing by WADA, may choose rapid hyperhydration protocols, the effects of a shorter supplementation protocol on doping related blood parameters, warrants investigation. Therefore, a Gly excretion study, administrating the hyperhydrating solution containing Gly, Cr and Glu over the period of one day, was also conducted to relate hematological changes following supplementation with the hyperhydrating solution containing Gly to the pattern of Gly excretion. 

## 2. Methods

### 2.1. Subjects

Nine trained males (Mean ± S.D. Age: 32.0 ± 10.0 years, Height: 177.0 ± 7.0 cm, body mass (BM): 71.0 ± 6.0 kg; Aerobic Capacity (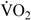
max): 61.0 ± 4.0 mL·kg^−1^·min^−1^) gave their written informed consent to take part in the present study that was approved by the Ethics Committee for Non-Clinical Research Involving Human Subjects, University of Glasgow and was performed according to the code of ethics of the World Medical Association (Declaration of Helsinki).

### 2.2. Study Design: Supplementation

Supplementation entailed ingestion of 1.0 g·kg^−1^ BM of Gly (Glycerin, Care plus, Huddersfield, UK), 10.0 g of CrH_2_O (Creapure Creatine Monohydrate, Reflex Nutrition Ltd., UK) and 75.0 g of Glu (SISGO Electrolyte Drink Powder, Ashwood Laboratories, Lancashire, UK). Participants were instructed to dissolve all ingredients of the supplement in approximately 20 mL of hot water (60–70 °C) and afterwards to add room temperature water (20–22 °C), to make total volume 1 L and consume supplement twice daily for 7 days. This supplementation protocol has been previously shown to successfully induce hyperhydration following 7 days of ingestion [4,5,13] and therefore was used in the current study to allow direct comparison of our findings with previous literature. It should be noted that the fluid ingested with the hyperhydrating substances was a standard volume and not relative to BM. Similarly, the amount of Cr and Glu contained in the supplement were not relative to BM since doses contained in this supplement have been shown to enhance muscle phosphocreatine levels within 5 days [14], while the Glu amounts contained in this supplement have shown to be central in stimulating the uptake of Cr by the skeletal muscle cells [15].

Participants were instructed to consume the first supplement in the morning before breakfast and the second one in the afternoon, with intervals being no more than 5 h, and were asked to consume supplements at the same time every day for the duration of the supplementation week. Participants reported to the laboratory, after an 8-h fast, on three occasions for measurements (familiarization test, pre- and post-supplementation test), all separated by one week. During the familiarization week, participants simply consumed 2 L of water/day in order to replicate anticipated fluid intake during the supplementation week. The supplementation period started on the day after the 2nd test and finished the day before the 3rd test. 

All supplements were made fresh before consumption to avoid degradation of Cr to creatinine [16]. Participants followed their normal diet and completed 7-day food diaries during the familiarization and pre-supplementation weeks and were asked to replicate their training practices throughout the study period. The diet was analyzed for energy intake and macronutrient content using the CompEat nutritional analysis software, which is based on a UK, integrated database (Nutrition Systems, Banbury, Oxon, England, UK) [17]. Participants were asked to avoid caffeine intake and alcohol for the full length of their participation in the study to lessen any possible confounding effects of caffeine on Cr [18]. 

### 2.3. Procedures

**Figure 1:**
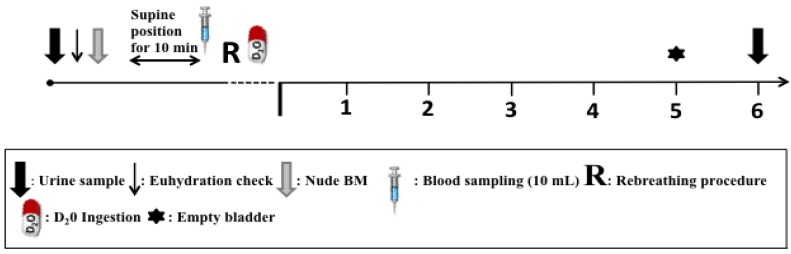
Schematic representation of procedures during experimental trials.

All procedures described below and shown schematically in Figure 1 were carried out on all visits to the laboratory (familiarization, pre- and post-supplementation) with the exception of the TBW measurement, which was only performed in the pre-, and post-supplementation trials. Measurements during the 1st visit to the laboratory (familiarization trial) were only performed to ensure that the participants were introduced to and were comfortable with all procedures and therefore data from familiarization trials were not reported. On arrival to the laboratory, participants provided a baseline urine sample before nude BM was recorded. Euhydration was confirmed, prior to the start of each experiment, by measuring urine osmolality and all subjects were found to be euhydrated (155 ± 55 mOsm/L). Following this, a 21 G cannula was introduced into a superficial vein of the anticubital fossa of the subject’s right arm. Blood samples (10.0 mL) were taken before and after a rebreathing procedure used to measure total Hb mass (tHb-mass) (see protocol below) [16,19,20]. Participants had adopted a supine position 10 min prior to each blood sampling as posture change induces PV shifts, which in turn can influence concentration based measures such as [Hb] and Hct [21] and PV derived from tHb, [Hb] and Hct. Participants were then asked to orally ingest 0.5 g·kg^−1^ BM deuterium oxide (D_2_O) in the morning after a baseline urine sample had been collected. D_2_O is a stable (nonradioactive) isotope of hydrogen, distinguished by its additional neutron. Once orally ingested, D_2_O mixes with body water and is eliminated from the body along with unlabeled water. The calculation of water intake from D_2_O elimination is based on the assumptions described by Fjeld *et al.* [22]. D_2_O for the present study was purchased from the Doubly Labeled Water (DLW) Resource Center, University of Aberdeen. Participants were instructed to empty their bladder completely at 5 h post D_2_O ingestion and in order to evaluate the isotopic decay in body water; a urine sample was collected again in a dry plastic container 6 h after ingestion of D_2_O. Participants were allowed breakfast, a light lunch and were allowed to pass urine and drink as normal within the 6 h period, as consumption of food and fluids during the 6 h period has been shown not to affect the post D_2_O measurement of TBW [23] For purposes of analysis, the investigator transferred 2.0 mL from all urine samples from the plastic containers to glass vessels and stored at −20 °C until analysis. Urine samples were then sent in one batch to the University of Aberdeen for isotopic analysis by an isoprime isotope ratio mass spectrometer (Isoprime Limited, Earl Road, Cheadle Hulme, Cheadle, UK) coupled to a Eurovector gas chromatatographer (GC; Eurovector, Via Torana, Milan, Italy) fitted with an HT300A autosampler (HTA, Via del Mella, Brescia, Italy) as previously described [24]. 

### 2.4. Blood Treatment and Analysis

Blood was drawn into dry syringes (Baymed Healthcare Ltd., East Kildbride, UK) and 2.5 mL dispensed into a 4.0 mL tube containing K_3_EDTA (Vacuette 4 mL K2EDTA, Greiner Bio-One, Stonehouse, UK). The K_3_EDTA tube was used to analyze Hb, Hct and Ret% by automated methods (ABL 725, Radiometer, Copenhagen, Denmark; Sysmex XT-2000i, Sysmex UK Ltd., Milton Keynes, UK).

### 2.5. Total Hemoglobin Mass (tHb-Mass) Analysis

**Figure 2:**
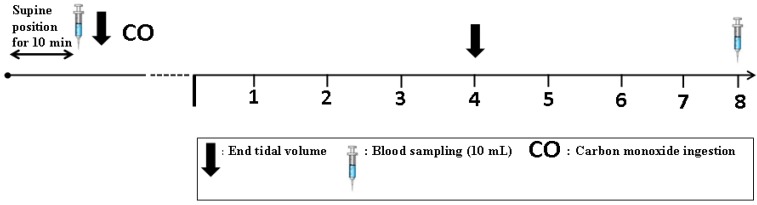
Schematic representation of tHb-mass procedure.

The optimized CO re-breathing method was used to determine tHb-mass pre- and post-supplementation and is shown graphically in Figure 2 [16,19,20]. Briefly, a bolus of chemically pure CO dose of 1.0 mL·kg^−1^ BM was administered with the first breath through a spirometer and rebreathed for 2 min with 4.0 L of oxygen. Change in percent carboxyhemoglobin in venous blood samples (from baseline to 8 min after CO administration), analyzed using a blood gas analyser (ABL 725, Radiometer, Copenhagen, Denmark), was used to determine tHb-mass. In addition, erythrocyte count as well as PV was derived as previously described elsewhere [25]. During pre- and post-supplementation tests, the optimized carbon (CO) monoxide re-breathing method was performed several times (without the use of CO) prior to the ‘real’ measurement. This was done to ensure that subjects were familiar and comfortable with the procedure and to avoid errors and leaks during the actual measurement. Work performed in our laboratory showed that typical error of tHb-mass measurement is <2% and is in agreement with previous findings [19]. Generally, the procedure was conducted in good manner with no leaks being detected during the real measurement and participants of the current study tolerated the procedure well with no signs of CO toxicity.

### 2.6. Blood Modeling Calculations

Indirect methods based on the statistical application of hematological parameters such as Hb, Ret% and tHb-mass have been developed to detect blood manipulation [26,27,28]. This study applied the OFF-hr model score [25] and Hbmr marker model (hybrid algorithm) [27] as determinants of altered blood profiles and their respective calculative descriptions are shown below: 

OFF-hr model score were calculated as previously described [26]:


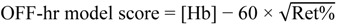


where [Hb] is in g·L^−1^, Ret% is percent reticulocytes.

Hbmr markers were calculated as previously described [27]:


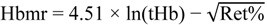


where ln(tHb) is natural log of tHb mass in g, Ret% is percent reticulocytes.

### 2.7. Gly Excretion Study

A Gly excretion study was conducted on a separate cohort of participants (*N* = 6; Mean ± S.D. Age: 25.5 ± 1.9 years, Height: 1.8 ± 0.1 cm, BM: 77.5 ± 12.4 kg) and presented here. Participants of the Gly excretion study consumed the supplement of the same composition as in the main study, but only for the course of one day; the supplementation period for participants commenced in the morning upon arrival at the laboratory and finished prior to leaving the laboratory on the same day. In summary, participants reported to the laboratory at 9 am on the experimental day whilst previously having refrained from food or water in the previous 8 h and alcohol and strenuous exercise in the previous 24 h. Participants were asked to void and euhydration was confirmed as in the main study (Average urine osmolality: 160 ± 52 mOsm/L). Nude BM was then recorded and TBW estimated using a Bodystat Bioimpedance analyser (BIA; Quadscan 4000, Bodystat Ltd., Isle of Man, UK). The bioimpedance measurements were taken while the subjects lay comfortably in a supine position for 10 min on a nonconductive surface with their arms and legs slightly abducted. Briefly, BIA is a non-invasive method that involves placing two current-inducing electrodes and two detector electrodes on the dorsal surfaces of the right hand and foot and a small (and imperceptible) electrical current (500 Micro-Amps) introduced between these. Following the estimation of TBW with the use of BIA, a 21G cannula was introduced as described in the main study and a baseline blood sample (2.5 mL) drawn. Immediately after the first blood sample the subject was asked to consume the first 1 L of the hyperhydrating solution containing Gly within 15 min. Following supplementation of the hyperhydrating solution, a 2.5 mL blood sample was collected every 15 min (Gly appears in the circulation within 15 min of supplementation [29]) for a 90-min period (when blood Gly concentration is expected to peak following supplementation [29]) and every 30 min for 6.5 h thereafter. The line was kept patent with a 2.5 mL flush of isotonic saline after each blood sample was taken. The second supplement was subsequently consumed 4 h after the first supplement, as this represented enough time for blood glycerol to return to resting levels [29]. 

Participants provided urine samples at baseline and at least every 2 h for 8 h on the day of the experiment and continued providing samples every 2 h up to 24 h. Participants were provided with plastic containers in which they passed urine and were advised to label each container (*i.e.*, sample 1, 2, *etc.*). Participants were also advised to complete a diary in which they recorded the sample number and the time and volume of urine. Urine was primarily collected in a measuring cylinder and then a representative sample decanted into the plastic containers. Participants were advised to keep plastic containers cool in the cool bag provided and transfer them to the freezer at the end of each day. Finally, BM and TBW were recorded at the end of the experimental day and in the morning of the following day when participants were asked to report to the laboratory before breakfast. Participants were asked to refrain from eating or drinking during the 8 h of the experiment with the exception of the hyperhydrating solution containing Gly. Participants were advised to have their light dinner after the experiment was finished and for at least 8 h before the following morning. Blood was collected and analysed for Hb and Hct as previously described in the main study. Changes in Hb and Hct relative to initial baseline values were used to calculate PV changes [30]. Blood modeling calculations were also carried out as previously described. Urinary [Gly] was assessed using the method described by Thevis *et al.* [31] on a Agilent 6890 gas chromatograph coupled to an Agilent 5973 mass spectrometer (both Agilent, Waldbronn, Germany). The limit of quantification was 0.9 μg·mL^−1^. Samples exceeding the working range (0.9–96.0 μg·mL^−1^) were diluted and re-analyzed. In order to account for the volume dependent urine dilution, raw urinary [Gly] (C_raw_) were corrected to a specific gravity of 1.02 g·mL^−1^ according to equation mentioned elsewhere [8]. Urine specific gravity was measured on DMA 38 Density Meter (Anton, Paar, Austria). 

### 2.8. Statistical Analysis

Data were assessed for normality of distribution and descriptive analysis was carried out to reveal the Mean ± S.D. Given the relatively small sample size, data were also examined via non-parametric procedures such as the Mann Whitney and Kruskal-Wallis tests. The non-parametric tests agreed with the findings of the parametric tests and therefore only the parametric test results are presented. Paired *t*-tests were used to examine differences between pre- and post-supplementation (main study). The Gly excretion study employed repeated measures ANOVA used to examine differences between baseline and each time point of observation. All statistical analysis was carried out using SPSS (Statistical Package for the Social Sciences; SPSS Inc., Chicago, Illinois) for Windows version 17.0. Statistical significance was set at *P *≤ 0.05. 

## 3. Results

### 3.1. Main Study

**Figure 3:**
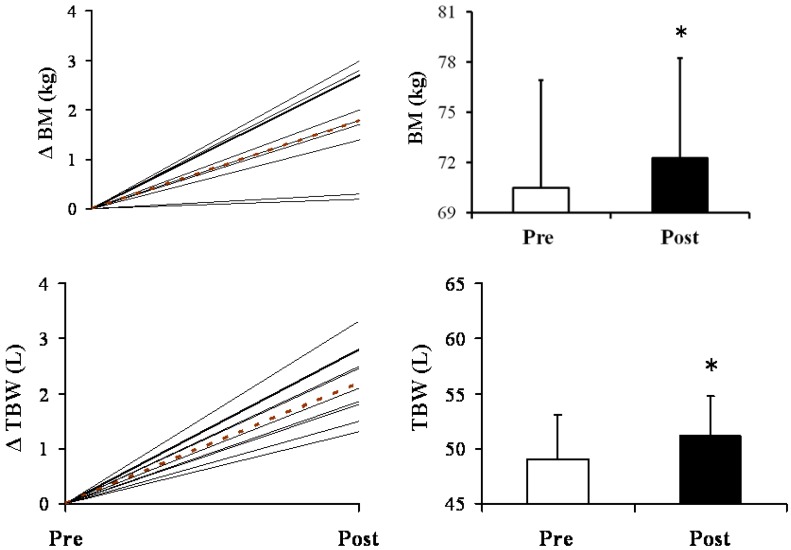
Changes in body mass (BM) and total body water (TBW) induced by 7-day supplementation with the hyperhydrating solution containing glycerol (Gly), creatine (Cr) and glucose (Glu). Dashed lines represent mean change of the group and solid black lines represent individual data. Bar charts show Mean ± S.D. values of BM and TBW pre- and post-supplementation, *N *= 9. * Significant (*P *< 0.01) difference between pre- and post-supplementation.

BM and TBW changes are presented in Figure 3. BM and TBW increased significantly following supplementation by 1.1 ± 1.2 and 1.0 ± 1.2 kg (BM, *P *< 0.01; TBW, *P *< 0.01; Figure 3), respectively. 

**Figure 4:**
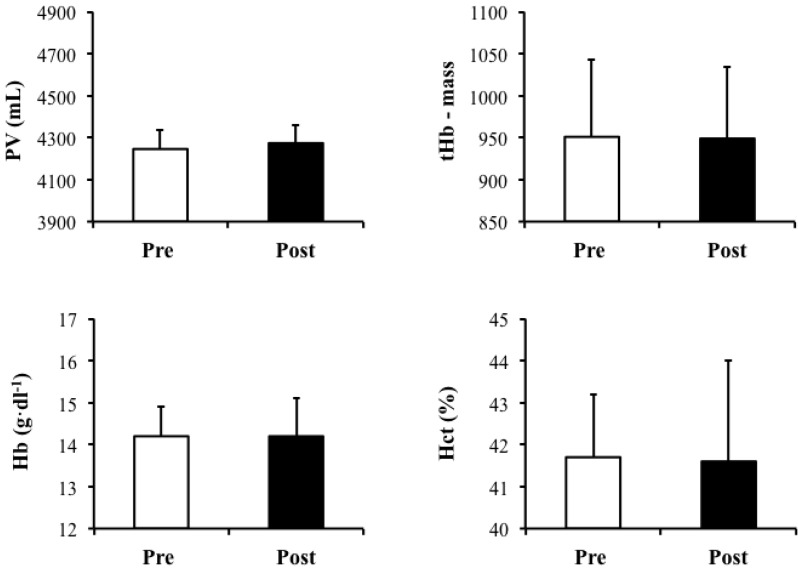
Plasma volume (PV) (mL), total Hb mass (tHb-mass), total hemoglobin (Hb) (g·dL^−1^) and hematocrit (Hct) (%) pre- and post-supplementation with the hyperhydrating solution containing Gly, Cr and Glu. Data presented as Mean ± S.D., *N *= 9.

In order to provide an overview of the effects of 7-day supplementation with the hyperhydrating solution containing Gly, Cr and Glu on blood parameters, pre- and post-supplementation values of PV, tHb-mass, Hb and Hct are presented in Figure 4. PV (Pre: 4246.6 ± 424.0 mL, Post: 4274.1 ± 457.7 mL; *P *= 0.7), tHb-mass (Pre: 936.1 ± 98.6 g, Post: 933.2 ± 94.2 g; *P *= 0.8), Hb (Pre: 14.3 ± 0.7 g·dL^−1^, Post: 14.3 ± 0.9 g·dL^−1^; *P *=0.9) or Hct (Pre: 41.9 ± 1.5%, Post: 41.7 ± 2.3%; *P *= 0.8) were not significantly different between pre- and post-supplementation. 

Table 1 presents the effect of the supplementation with the hyperhydrating solution containing Gly, Cr and Glu on indirect doping markers (Ret%, the OFF-hr Score and Hbmr). Values of Ret%, OFF-hr score and Hbmr were not significantly altered by supplementation. 

**Table 1:** Reticulocytes (Ret%), OFF-hr score and hybrid algorithm (Hbmr) values, pre- and post-supplementation of the hyperhydrating solution containing Gly, Cr and Glu. Data presented as Mean ± S.D. and mean differences (95% CI) of different hematological parameters calculated from pre- and post-supplementation values, *N* = 9.

Doping Markers	Pre	Post	Mean Difference
Ret%	1.0 ± 0.3	1.2 ± 0.4	−0.2 ± 0.3
OFF-hr score	83.4 ± 9.4	75.5 ± 9.1	−7.9 ± 4.0
Hbmr	29.9 ± 0.5	29.8 ± 0.5	−0.1 ± 0.1

### 3.2. Data from Gly Excretion Study

**Figure 5:**
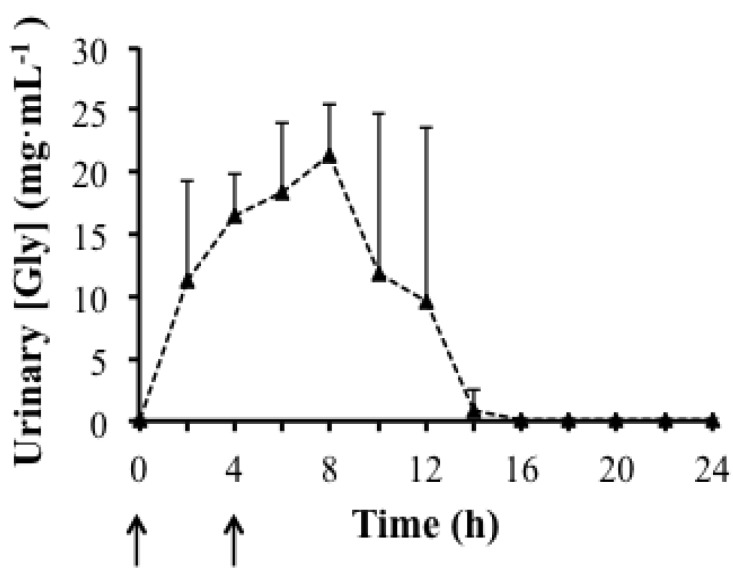
Mean ± S.D. values of urinary excretion of Gly (mg·mL^−1^) pre- (0 h) and post-supplementation (2–24 h) of the hyperhydrating solution containing Gly, Cr and Glu. *N* = 6; ↑: ingestion of 1st and 2nd solution at 0 h and 4 h, respectively.

**Figure 6:**
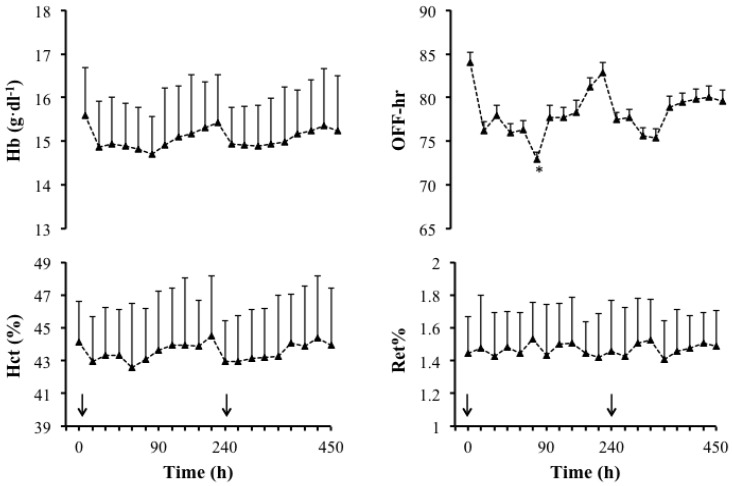
Mean ± S.D. values of Hb concentration (g·dL^−1^), Hct (%), OFF-hr score and Ret% pre- (0 h) and post-supplementation (2–24 h) of the hyperhydrating solution containing Gly, Cr and Glu. *N* = 6, ↓: ingestion of 1st and 2nd solution at 0 h and 4 h, respectively. * Significant (*P* < 0.05) difference between pre- and post-supplementation.

BM was significantly higher at the end of the experimental day and in the morning of the next day compared to baseline; 0.4 ± 0.1 and 0.6 ± 0.4 kg, respectively (*P *< 0.01). Conversely, TBW measured with BIA was not significantly affected by supplementation (ANOVA, *P *= 0.1). Figure 5 shows the pattern of Gly excretion following the supplementation. Before supplementation, urinary [Gly] was very low and similar in all subjects and ranged from 0.0 to 0.2 mg·mL^−1^. Urinary [Gly] increased from 0.0 ± 0.1 mg·mL^−1^ (0 h) to 11.2 ± 8.1 mg·mL^−1^ in the first urine sample (2 h) and 16.4 ± 3.5 mg·mL^−1^ in the final sample taken after supplementation of the first solution (4 h). At 4 h, a second drink was administrated and urinary [Gly] increased further to 18.4 ± 5.6 mg·mL^−1^ in the next urine collection (6 h). Urinary [Gly] peaked at 8 h (21.3 ± 4.1 mg·mL^−1^) and then started decreasing gradually towards baseline. Urinary [Gly] returned to baseline (0.0 ± 0.0 mg·mL^−1^) after 16 h in all subjects. Figure 6 shows responses of blood markers pre- and post-supplementation. Despite the urinary changes in [Gly] over time, concentrations of Hb, Hct and Ret% were not significantly different between baseline and any other time point (ANOVA, Hb: *P *= 0.3; Hct: *P *= 0.3; Ret%: *P *= 0.9). The OFF-hr score was significantly lower after 75 min of observation compared to baseline (*P *= 0.02). PV changes calculated using values of Hb and Hct measured at baseline and at each time point of observation (assuming no change in red cell mass during the supplementation period) increased by ~2.7% at the end of the supplementation day but this increase was not significantly different (*P *= 0.9).

### 3.3. Side Effects

In general, subjects tolerated the supplementation protocol well, with only 1 report of light headaches after the supplementation protocol of the Gly excretion study. No side effects were reported following the supplementation protocol of the main study.

## 4. Discussion

The main aim of the current study was to determine whether supplementation with a hyperhydrating solution containing Gly, Cr and Glu for 7 days leads to significant differences in PV and markers of blood manipulation such as Hb, Hct, tHb-mass, Ret%, OFF-hr score and Hbmr, currently used to identify doping. Despite an increase in TBW, PV and the aforementioned blood-relevant doping markers were not significantly influenced by supplementation with the hyperhydrating solution containing Gly, Cr and Glu. In addition to the main study, we conducted an observational study on urinary Gly excretion and doping-related blood measures following consumption of the hyperhydrating supplement. Despite a significant increase in BM over the course of supplementation period lasting 8 h, PV changes and the blood-relevant doping markers of interest were not significantly affected.

It is not unexpected that PV estimated before and after 7 days of supplementation with the hyperhydrating solution containing Gly, Cr and Glu was not significantly affected, as is has previously been shown that such supplementation leads to PV maintenance rather than PV expansion [4,5]. In the Gly excretion study we found that following supplementation with the hyperhydrating solution containing Gly, urinary [Gly] levels exceeded the renal threshold (0.2 mg·mL^−1^; Figure 5) that has recently been proposed [31]. This threshold was established by assaying a total of 1039 doping control urine samples found to be “negative” and collected from elite athletes to provide reference data for “normal” concentrations found in “in-competition” as well as “out-of-competition” samples of various sport disciplines [31]. The extensive analysis conducted on the doping control urine samples proposed a “safe” threshold level, representative of typical dietary practices, to be considerably lower than 0.2 mg·mL^−1^[31]. 

The Gly supplementation used in the present study raised urinary [Gly] levels well above this threshold and to levels considered by some with potential to expand the PV; albeit no significant PV expansion was found in the present study. Although circulating [Gly] was not measured in the present study, it would appear from the measured urinary [Gly] (assuming a link between plasma and urine Gly), that peak plasma concentrations between 10 and 20 mmol/L [29,31,32,33,34] were reached following supplementation. Therefore, the potential exists for osmotic forces resulting from the markedly elevated plasma [Gly], to move water into the vascular space and expand blood and plasma volume [27]. Although this study is the first to have measured the hyperhydrating effects of the Gly hyperhydrating solution by measuring TBW using the gold standard technique of D_2_O ingestion, measurements of ICW and ECW were not estimated. Other studies, however, which have used exactly the same hyperhydrating supplement containing Gly, have reported non-significant changes in PV between pre- and post-supplementation despite significant increases in TBW, ICW and ECW [4,5].

The finding of no significant expansion in PV following the ingestion of the hyperhydrating solution containing Gly in the main and Gly excretion study as well as other studies [4,5] is not surprising given the limited potential for PV expansion in relation to total PV [35]. Indeed, in the main study we found, on average, a 1.0 L increase in TBW and corresponding 28 mL (1.0% increase) PV expansion estimated from the direct measurement of tHb-mass via the optimized CO method [16]. In the Gly excretion study PV change of 2.7% (135 mL) was found using Dill and Costill method. These results are consistent with studies reporting no significant differences in Hb and Hct following Gly hyperhydration; a reflection of the non-significant PV expansion. For example, two studies estimating PV changes following Gly supplementation, as estimated by using the Dill and Costill method [30], found an increase of ~78 mL [30] and 120 mL [36], from baseline to the end of the experimental day. Furthermore, given that glycerol is distributed throughout the entire TBW, the 2 L of fluid consumed with supplement will also be distributed evenly throughout the entire TBW. Given that TBW is ~2/3 ICW and 1/3 ECW then ~1.3–1.4 L can be expected to be added to the ICW and ~0.6–0.7 to the ECW. Since PV is ~1/4 of ECW (while ISF is ~3/4) an increase in PV of 150–170 mL can be expected. Such insignificant PV expansion is unlikely to have any masking effect. Other studies, however, using the indirect Dill and Costill method [30] to assess PV changes report significant increases in PV of 7.5% [32] and up to 10.0% [33] following Gly (1.0–1.1 g·kg^−1^) ingestion. Assuming a 70 kg male has a typical blood volume of 5 L, of which ~55.0% is PV, in the above studies, would have enhanced PV by ~225–300 mL. Difference between studies could stem from different protocols being employed in terms of timing of ingestion and different methods used to estimate PV changes as well as timing of measurements and therefore, more studies employing comparative protocols and more direct “gold-standard” methods to estimate PV expansion such as the method used in the current study should be carried out.

There is some evidence to suggest that PV expansion may simply reflect fluid ingestion rather than be related to hyperhydrating qualities of Gly included in supplement. For example, in a study where change in PV was estimated following both supplementation with Gly (1.0 g·kg^−1^ BM) or placebo, following Gly supplementation PV was increased by up to 5.0% (estimated at 150 mL assuming no changes in red cell mass occurring from baseline and that total PV is ~3 L) and following placebo ~2.2% (60 mL), with differences between treatments being not significant [37]. Similarly, a recent study funded by WADA [8] reported a similar PV change following 1.0 g·kg^−1^ BM of Gly compared to water ingestion alone (*i.e.*, 7.5% or equivalent to ~225 mL compared to 4.5% or equivalent to ~135 mL) [8]. In the present study we administered 1.0 L of water (*i.e.*, ~14 mL·kg^−1^ BM) with each hyperhyrdating solution containing 1.0 g·kg^−1^ BM Gly, which is similar to studies administrating 22.0–25.0 mL·kg^−1^ BM of water (with the Gly solution) [8,37]. We further compared our findings with other studies in the literature [33,38] that although did not have as a primary aim to induce hyperhydration, provide detailed results on PV following Gly administration. It seems that we have administrated higher amounts of water with each Gly solution, compared to these studies that have administrated water equaled to 225.0 [33] and 400.0 mL [38]. Nevertheless, the present study and studies by others [8,34] administrating higher volumes of water per each Gly intake, did not find a significant difference in PV following supplementation. 

In the present study, the hyperhydrating solution consisted of Gly, Cr and Glu and therefore, it could be argued, that addition of Cr and Glu could have attenuated the increase in PV. However, it is unlikely that the addition of Cr to the Gly supplementation mixture could have diminished the plasma expansion potential of Gly since this combined Cr and Gly supplementation protocol has previously been shown to result in a greater TBW increase (0.9 L) as opposed to Gly alone (0.5 L). Interestingly and in agreement with the present study, this greater increase in TBW following Cr and Gly supplementation did not significantly alter PV or indeed the hematological parameters, Hb and Hct (%) measured [4]. 

The current study found that 7-day supplementation or supplementation over 8 h with a hyperhydrating solution containing Gly did not induce differences in Hct (%) or Hb and this is in agreement with previous findings [4,5]. Similarly, we did not detect any differences in the indirect doping markers such as tHb-mass and Hbmr or Ret%. Total Hb-mass can be used as an alternative method for long-term identiﬁcation of supraphysiologically elevated Hb levels [39], while the novel Hbmr marker proposed by Mørkeberg and colleagues (2011) has the advantage of incorporating both tHb-mass and Ret% [27]. Both parameters are fairly stable and less amenable to dilution and tampering. For example, Ret% represents the ratio between the concentration of mature erythrocytes and immature erythrocytes in a sample and therefore is not subject to modification by changes in PV changes (either naturally or manipulated) [27]. This resistance of tHb-mass and Ret% to PV change is in contrast to the OFF-hr score, which is less resistant to PV changes [40]. Nevertheless, supplementation with the hyperhydrating solution containing Gly in the present study did not significantly alter the OFF-hr score. Collectively, the present data fail to support the idea that Gly can induce a level of PV expansion necessary to significantly alter routinely used doping-relevant blood parameters even when Gly is ingested at levels above those resulting in peak plasma [Gly] concentrations [29,32,33,34,41]. It should be noted that during the 7-day supplementation regime, participants were required to be fasted 8–12 h prior to attending the laboratory, and thus the final solution containing Gly, Cr and Glu would have been consumed ~12 h prior to the tHb-mass measurement from which PV is estimated. It has been shown that unless continued dose of Gly is given to offset urinary Gly excretion and metabolism of Gly, hyperhydration cannot be maintained [42]. Arguably, therefore, this protocol could eliminate any opportunity to detect an effect of supplementation on PV. However, in the Gly excretion study, blood measurements were taken every 30 min up to the end of the 8 h period, following the consumption of the final supplement containing Gly, Cr and Glu and can confirm that the absence of significant differences in hematological parameters following Gly, Cr and Glu supplementation, are not due to an incorrect timing of measurements. Similarly, the fact that, in Gly excretion study, there was no difference in TBW measured at baseline and in the morning following the experiment, could be related to increased excretion of urine experienced by participants overnight and therefore to the timing of the measurement. On the other hand, inability to detect TBW changes may be related to the use of BIA rather than D_2_O in the Gly excretion study. Indeed, we have recently showed that the method used to detect TBW changes in the Gly excretion study does not correlate with the gold standard method of D_2_O [13]. Therefore, changes in BM rather than TBW changes were used to evaluate hyperhydration.

## 5. Conclusion

This study indicates that a Gly supplementation regime designed to maximize hyperhydration by co ingesting Cr and Glu, does not lead to PV expansion and consequently, has no significant effect on the examined hematological profiles and therefore of limited or no potential to act as a masking agent of doping related blood parameters. A well-controlled study administrating relevant doping-related substances in combination with the present hyperhydrating supplement containing Gly, could be carried out to confirm the findings of the present study.
